# PrEP Among Sub-Saharan African Diaspora Communities in Belgium – a Participatory Action Research Study

**DOI:** 10.1007/s10900-023-01269-7

**Published:** 2023-08-14

**Authors:** Ella Van Landeghem, Alida Arbier, Christian Sydney A. Pratt, Mikaza Senga, Gert Scheerder, Thijs Reyniers, Bernadette Hensen, Christiana Nöstlinger

**Affiliations:** 1 Department of Public Health, Institute of Tropical Medicine, Nationalestraat 155 Antwerp, Antwerp, 2000 Belgium; 2 Department of Clinical Sciences, Institute of Tropical Medicine, Antwerp, Belgium

**Keywords:** PrEP, HIV, Qualitative, Sub-Saharan African, Participatory action research

## Abstract

In Belgium, migrants from Sub-Saharan Africa (SSA) accounted for 45% of new heterosexual HIV infections in 2021, while only 1.5% of PrEP starters were of SSA descent. We explored the acceptance of PrEP and barriers towards PrEP uptake and use among SSA migrant and diaspora communities in Belgium using a participatory action research approach. Trained community researchers (CRs), involved in all phases of the study, co-designed and moderated group discussions (GDs) while simultaneously providing information on HIV and PrEP during workshops. Extensive summaries and field notes were analysed using reflexive thematic analysis. CRs were involved in data analysis, interpretation and reporting. We conducted seven GDs with 51 participants. We identified five major themes: (1) Participants had limited PrEP knowledge, which created feelings of surprise and annoyance about not being informed. This was partly explained by (2) the taboo and stigma that surrounds sexuality and HIV, which could shape PrEP acceptance. (3) Participants shared feelings of otherness due to experiences of racism and discrimination, also in relationship to HIV prevention. (4) PrEP was considered a high-threshold prevention tool, because of its perceived side-effects and its specialized service delivery. (5) Despite nuanced opinions about PrEP, all participants agreed that PrEP promotion should be mainstreamed, so everyone can make an informed decision. In conclusion, PrEP seemed acceptable among our participants. Our qualitative study provides insights into the intersecting barriers to accessing HIV services, showing that SSA diaspora communities are ‘hardly reached’ rather than ‘hard to reach’ by PrEP promotion messages.

## Introduction

HIV remains a major public health concern in Europe and Belgium [1, 2]. To achieve the 95-95-95 targets set by the Joint United Nations Programme on HIV/AIDS (UNAIDS), a renewed focus on primary prevention is needed [3]. Pre-exposure prophylaxis (PrEP), i.e. the preventive use of oral antiretroviral medication, is a highly effective HIV prevention tool. Among 55 countries reporting to the European Centre for Disease Prevention and Control (ECDC), 36 provide PrEP through their national health services. Among them, 22 also reimburse PrEP, either through health insurance or the public sector [4]. However, disparities in PrEP uptake exist across Europe. While PrEP is being used by increasing numbers of men who have sex with men (MSM), other affected populations including women, migrants of high endemic regions and sex workers are hardly being reached [5–8].

The Belgian HIV epidemic is characterized by two concentrated epidemics among MSM and individuals with a Sub-Saharan African (SSA) nationality [2]. In 2013, a community-based survey showed that HIV prevalence among SSA migrants in Antwerp was 5.9% among women and 4.2% among men [9]. In 2021, 45% of all newly reported heterosexual diagnoses were among individuals with a SSA nationality, although in this proportion a downward trend has been observed over recent years [2]. Additionally, a study in nine European countries in 2015, using clinical data and a cross-sectional questionnaire, estimated that about 45% of SSA migrants living with HIV acquired HIV post-migration [10]. This evidence suggests that a renewed emphasis on primary prevention among this underserved population is needed.

In 2017, Belgium became one of the first countries in Europe to deliver PrEP through a national reimbursement scheme, using specific eligibility criteria [11]. Currently, PrEP is delivered through specialized HIV clinics. Once users are initiated on PrEP, follow-up visits are required every three months [11]. Despite being disproportionately affected by HIV, migrants from SSA and men having sex with men with a migrant background experience barriers to take up PrEP in several Western-European countries [12]. This is in line with the Belgian experience, showing that that only 1.5% of individuals initiating PrEP in 2021 were nationals of a Sub-Saharan African country. Clearly, the number of PrEP users with a migrant background does not correspond to the composition of the newly reported HIV diagnoses in Belgium [2].

Limited research has looked into the acceptance of PrEP among SSA diaspora communities. Existing studies differ in study population, ranging from black gay, bisexual and other MSM [13] to SSA migrants in high income countries [14]. The most common barriers to accessing and using PrEP among minoritized populations include a lack of awareness and information about PrEP [12, 14–19], stigma due to its potential association with HIV [5, 12, 14, 17–21], a reluctance to discuss sexual health [14, 17–20] and low HIV risk perception [12, 14, 17, 18, 21]. Among migrant populations, legal status, financial and residential instability and accompanying competing priorities to mitigate this precariousness can also limit PrEP uptake [14, 16, 18, 19]. Furthermore, a centralized PrEP service delivery model, such as in Belgium, may not be adapted to migrants’ needs [22], or is perceived as something targeting mainly white gay men [14, 18, 23].

Despite this limited evidence, calls have been launched to improve PrEP uptake among sub-Saharan African diaspora [17], evidence on the acceptance of PrEP among SSA communities is limited, especially in Belgium [14]. With this study, we aim to gain insights for the Belgian context into the acceptance of PrEP and barriers towards PrEP uptake and use among Sub-Saharan African migrant and diaspora communities in Belgium.^1^

## Methods

### Study Design

#### Theoretical Framework

This inductive qualitative study used a participatory community-based action research (PCBAR) approach, involving community members in all phases of the research process [24, 25]. In a preparatory phase community members expressed a need for more information on PrEP. Therefore, we deviated from traditional focus group approaches to action research. We chose group discussions (GD) as a data collection method to allow insight into the community perceptions towards PrEP [26]. We selected and trained community researchers (CR) to co-design the topic guide and moderate GDs, while providing information about sexual health and PrEP in a workshop format. CRs were involved in final analyses and writing up of the results.

#### Participant and Community Researcher Recruitment

We recruited CRs during a formative research phase of a research project on PrEP implementation, [27] and through existing pre-established HIV prevention networks. CRs were selected based on identifying as member of a Sub-Saharan African diaspora and experience in the field of sexual health and HIV prevention. CRs were trained in sexual health, including HIV and PrEP, and qualitative research methods, with a specific focus on GD.

Study participants were recruited through CRs’ personal and professional networks using social media, personal connections with asylum reception centres and other community-based organisations. We intentionally kept inclusion criteria broad and open due to the explorative nature of the study. The two inclusion criteria for study participants was to self-identify as a member of the diaspora of a Sub-Saharan African country and to be able to give written informed consent to participate. Some participants self-identified as a member of these communities despite not being born in a sub-Saharan African country. In collaboration with our community researchers, we aimed for diversity of participants in GDs, and later on within these GDs. Therefore, we chose to keep some GDs homogeneous according to gender, age and sexual orientation, while also mixing these characteristics within other GDs.

### Data Collection and Action Research

Data were collected between May and June 2022 in confidential environments, either at a meeting room in a public health institute, or in gathering rooms of community-based organizations in the cities of Antwerp and Brussels. Participants filled in a socio-demographic questionnaire with open questions at the start of each GD. We developed a semi-structured topic guide in collaboration with community researchers. Topics included participant’s views on PrEP as an HIV prevention tool, the meaning they attribute to PrEP use, and whether and under what conditions PrEP might be beneficial for them and members of their communities.

We adopted a contextual approach, discussing sexual health in general before discussing PrEP. This was combined with delivering information on sexual health, giving participants the opportunity to ask questions and learn about PrEP within a safe space. Both moderators (AA, MS or CP) and observer (EVL) took field notes. GDs were conducted in multiple languages (French, English and/or Dutch), were audio-recorded, and extensive summaries were made in the language of the GD. Quotes were translated to English by the first author.

### Data Analysis

Summaries and field notes were analysed using a reflexive thematic analysis approach supported by NVivo 12 [28]. After familiarizing ourselves with the data, by re-reading the summaries and field notes, we coded the entire dataset, aiming to identify themes that answered our research question. Subsequently, we developed broader meaning-based patterns between these codes to generate initial themes. These were checked against the coded data and collaboratively discussed with the CRs to develop a more nuanced reading of the data. Before finalizing the analysis, we moved back and forth between the different steps, enabling us to refine, define and name the final narratives related to each theme. Analysis was not focused on finding consensus among all members of the research team. Where interpretations differed, we mention this in the results [28].

## Results

### Participants

We conducted seven GDs with 51 participants, with relatively equal distribution of self-identified gender (Table 1). Most of participants were single and had completed at least secondary education. Time lived in Belgium varied between eight months and 35 years, which, for some meant they were born in Belgium. The average age was 36,5 years. Table 2 describes discussion group composition. One GD included only women, one only men, one only young people (less than 25 years old), one only with people who identify as lesbian, gay or bisexual. The remaining GDs were composed of participants of mixed ages, genders and sexual orientations, and are referred to as mixed groups. Countries of origin were not defined as countries of birth, allowing for self-identification. For some this may not have been a Sub-Saharan African country (i.e. Caribbean or North-African), but these participants identified as African nonetheless.

**Table 1:** Participant characteristics

Socio-demographic factors	N = 51
Gender	
Female Male	26 25
Age	
< 25 26–50 50+ Not disclosed	12 28 9 2
Sexual orientation	
Heterosexual Lesbian, gay or bisexual (LGB) Not disclosed	35 10 6
Current relationship status	
Single Relationship Married Widowed Not disclosed	27 3 13 1 7
Education*	
Primary education Secondary education Higher education Not disclosed	1 24 12 14
Occupational status	
Student Employed Homemaker Unemployed Not disclosed	7 29 2 3 10
Years living in Belgium	
0–5 6–10 11–20 21–35 Not disclosed	10 8 13 16 4
Countries of origin	
Democratic Republic of Congo The Gambia Burundi Mauritania Senegal Cameroon Rwanda Other**	20 9 4 4 3 2 2 7

* Higher education is defined as education beyond secondary school

**Other countries of origin: Sierra Leone (n = 1), Mali (n = 1), Angola (n = 1), Tanzania (n = 1), Ivory Coast (n = 1), Aruba (n = 1) and Belgium (n = 1)

**Table 2:** Discussion group composition

	GD 1 (n = 9)	GD 2 (n = 8)	GD 3 (n = 5)	GD 4 (n = 8)	GD 5 (n = 9)	GD 6 (n = 5)	GD 7 (n = 7)
Gender							
Female Male	9 0	5 3	3 2	4 4	1 8	4 1	0 7
Age							
< 25 26–50 50+ Not disclosed	1 5 2 1	8 0 0 0	0 1 4 0	0 8 0 0	1 8 0 0	0 1 4 0	2 4 1 0
Sexual orientation							
Heterosexual Lesbian, gay or bisexual Not disclosed	9 0 0	8 0 0	3 0 2	0 8 0	7 2 0	2 0 3	4 0 3

### Main themes

#### “It is like they are hiding it” - PrEP Knowledge and information

The degree of knowledge on HIV transmission and treatment varied. Older participants described the effects of HIV/AIDS in the countries where they were born at the early stages of the epidemic, which had instilled a fear of the virus. Some participants had comprehensive HIV knowledge, however, many were not informed about recent developments in HIV treatment (e.g. the use of treatment as prevention). In some GDs, the discussion revolved around the terminology being used.

It’s the same thing with me, I only knew AIDS, not HIV. I thought the whites called it HIV, and we called it AIDS

Participant in GD 1 (female group).

Few participants personally knew someone who was living with HIV, which according to our participants could explain why their knowledge was limited.

In [country of birth] I only heard people talk about it, but I never saw it for myself. I never see somebody who has it. I do not know a lot about it, because I don’t experience it directly

Participant in GD 7 (male group).

The majority of participants heard about PrEP for the first time during the GDs. For some, this evoked annoyance and they expressed feelings of being forgotten and left behind.

No one knows about it because it’s not publicised, it’s not shared. It’s like they are hiding it, it’s as if they don’t want everyone to know about it

Participant in GD 1 (female group).

However, participants agreed that being informed about PrEP would be the first step into a higher acceptance of PrEP.

I think the first question will be, why would you use it, what’s the reason?” (…) “But I think with this knowledge, they [community] might at least consider it, or think more positive about it

Participant in GD 2 (young people group).

Participants largely agreed that there were several remaining uncertainties concerning PrEP, and had many questions throughout the GDs. The main topic of discussion was whether PrEP was intended for HIV negative individuals, to prevent one from getting HIV, or for HIV positive individuals to prevent onward transmission, showing some confusion with treatment as prevention (TasP) or post-exposure prophylaxis (PEP).

I have heard about PrEP before (…) from my understanding, PrEP is for people who have HIV and to not transmit it to people

Participant in GD 4 (LGB group).

**“It is always the same” - Stigmatization of African diaspora communities**.

While our participants described a feeling of being left out of PrEP promotion, some participants expressed a sense of stigmatization of the African diaspora when it concerns HIV, when African people are always singled out.

“Why always this kind of workshops, it is always the same? Always focused on African migrants. It is really not done in the right way (…) Where are the Arabs, the Latinos, the Asian people?”

Participant in GD 6 (mixed group).

Opinions on the importance of racism and discrimination and how much it influenced people, differed among community researchers. Noticeably, mainly the younger participants referred to the experiences of being black in a predominantly white country. They talked about experiences of racism and discrimination which they considered as one of the main challenges they were confronted with.

Of course, I also talked about it recently with friends. In the sense of, as you [other participant] say: I, for example, to this day, when I see police I feel scared. Whereas a white person who just feels safe when they see police. I think that’s because of different circumstances, of course. There is a very big difference between how you and they [white people] then experience those things

Participant in GD 2 (young people group).

#### “We cannot speak openly about it” – Taboos and stigma

According to participants, PrEP knowledge might also be shaped by community norms. Participants explained that such norms dictated that sex should happen within the boundaries of marriage, and that fidelity is important. Furthermore, participants shared that people should show constraint and abstain from sexual intercourse before marriage. Participants described that this was also closely linked to the stigma related to HIV, with the perception that an HIV positive diagnosis would imply sex outside of or before marriage. In combination with misconceptions regarding HIV transmission and treatment – this resulted in fearing HIV infection. For participants, HIV still implied a ‘death sentence’. Additionally, they described that it provoked feelings of shame, hindering HIV disclosure, as even families might abandon them. This was discussed as a barrier to discussing HIV prevention and PrEP uptake in their personal environment.

It’s also linked to the culture of societies, especially in Africa. When we say HIV/AIDS, it means that, directly, we see that you are sexually extravagant. So that’s it, that’s the problem

Participant in GD 3 (mixed group).

Additionally, participants feared confusion between HIV treatment and PrEP by other people. PrEP might be assumed to be taken as a treatment for HIV, which could fuel gossip. Using PrEP would imply sexual behaviour outside of marriage, hence it is possible PrEP users would suffer the same stigma as people living with HIV, as shown by the following excerpt:

Community Researcher: “How would other people react when they hear someone takes PrEP?”

[laughter among all participants]

*Participant: “I could not tell anyone, when I would they would ask: ‘what is this?’ and I cannot tell them that”*

Participant in GD 1 (female group).

Upon a better understanding of how PrEP works, some participants felt that PrEP did not fit with these descriptive sexual norms and could even encourage sex before marriage. However, they also described that in practice, sexual behaviours often differed from these injunctive norms. In such cases PrEP could be very beneficial, e.g. for sex workers or relationships where one partner might be unfaithful.

But when you would say it [PrEP] is for prevention, then it is just being responsible. The thing about your parents getting angry then, that’s more about them knowing you’re having sex

Participant in GD 2 (young people group).

Although participants described the younger generations as more open to talk about sexual health and relationships amongst each other compared to the older generation, they agreed that it was still difficult to talk about it with their parents. They described their parents as having conservative attitudes, which they attributed to their older age and socialisation in an African country. In contrast, older participants sometimes described the younger generation as “promiscuous”, looking at sex as a trivial matter. They partly blamed internet and social media for these attitudes, but younger participants also attributed this to their European upbringing or schooling.

*“Participant A: That [sexual health] is just not something you can just talk about at home. If I am honest, I would just get a beating.*

Participant B: They would say, you are not European, you are African!

*Participant A: I do have African roots, but I was born here and grew up here”*.

Participants in GD2 (young people group).

#### “It could be toxic for me” – Barriers to PrEP access and use

Concerns About PrEP

Participants expressed concerns about the side-effects of PrEP, as they frequently asked questions about this. Reactions tended to be negative when hearing there could be some, although very limited, side-effects.

For example, imagine if I meet someone new today. I’m like okay, I’ll take PrEP. It could be toxic for me, because I am healthy and all. Even if it is to protect myself.

Participant in GD 6 (mixed group).

Similarly, participants often wondered how effective PrEP really was in preventing HIV, referring to a sense of distrust in medical information from someone they do not know.

That’s why I asked if it worked 100%. There are so many medicines left and right, you can’t just start believing everything. So yes now, because I know you [community researcher] personally, I trust that you are not telling me lies. But if it were someone else…

Participant in GD 2 (young people group).

Participants added that this hesitance might be related to PrEP being a medication, which they tended to avoid, unless a person was ill. Most participants agreed that people from their communities would self-medicate before visiting a physician. The older generation tended to look for natural or traditional medicine first, as they had done in their country of birth. The younger generation were rather influenced by internet and social media when searching for information.

Our people also kind of avoid it, it is only discussed and taken care of when it gets really bad. They usually solve it by drinking ginger tea.

Participant in GD 2 (young people group).

### PrEP delivery

Some participants foresaw difficulties in accessing PrEP due to the way PrEP service delivery was organized. Some participants felt that this was in contrast to the idea of prevention, which should ideally be as low threshold and accessible as possible. As one participant highlighted, especially considering the spontaneity of sexual intercourse, one should be able to act quickly.

My concern is: they are doing prevention here, and prevention means to avoid something. That means that people are informed and... but then again, you are contradicting yourselves, you want to keep it as accessible as possible to inform people about it and to warn them that it exists and you can protect them from it, but on the other hand: before they get that medication, it’s quite an ordeal. That’s just... by then they have a partner and they are already HIV-positive. So I think that’s a bit of a contradiction, you know? And it might cost so much, people might not be able to afford it, you still have to qualify, to get a reimbursement, and it might not be sure that you qualify

Participant in GD 6 (mixed group).

### “It is for everyone” - Recommendations from participants

In spite of the anticipated difficulties regarding PrEP uptake and use, most participants found that using PrEP was a responsible choice, despite its connotations with sexuality and HIV. Especially among participants who identified as lesbian, gay or bisexual, it was considered well-suited for people who did not like using condoms.

I don’t mind using condoms, but some people might ask you to not use it, and in that case it might be a good idea

Participant in GD 4 (LGB group).

Overall, participants across all GD deemed prevention as important, emphasizing the need for accommodating personal preferences and granting free choice .

I agree with it [PrEP as something positive]. But I feel like if I was cautious in other ways, I could avoid taking the medication.

Participant in GD 5 (mixed group).

Generally speaking, participants agreed that more information on PrEP should be provided, as being informed was considered an important first step to better sexual health. They preferred this knowledge to be available to everyone, so everyone would be able to come forward for it.

I think it’s for everyone, it’s for everyone because you can’t say no, it’s for young people, for mothers. No, no, no, no, it’s for everyone

Participant in GD 1 (all female group).

Participants recommended that specific focus should be put on sex workers, young people getting ready for their *“hot girl summer”* (GD 2, young people group), people who have different sexual partners on a regular basis, people who do not like to use condoms, people who travel a lot or people who recently arrived in Belgium. However, participants agreed that everyone should have the right to choose if it is something that fits their needs and preferences.

Participants recommended to spread this information in community settings, such as women groups and schools. They also called for a better sexual health communication within the family. Using GDs and workshops such as the ones which were used for this data collection, flyers in mailboxes and posters in train stations, and using social media such as diaspora Facebook groups were deemed most suitable delivery channels.

## Discussion

In this study we gained insights into perceptions regarding the acceptance of PrEP and barriers to PrEP uptake and use among Sub-Saharan African diaspora in Belgium. During the discussions, we could identify five themes. First, knowledge and information on PrEP was limited. This is consistent with previous studies on SSA communities’ and PrEP [12, 14–19, 29]. Participants partly explained this by the taboo and stigma that rests on sexuality and HIV, but this non-awareness was also interpreted as being left behind, seen as confirmation of experiences with racism and discrimination. Once informed on PrEP, participants anticipated some barriers in accessing PrEP. Being a medication embedded within a specialized health care system it was seen as a high-threshold prevention method. Finally, participants also agreed that raising awareness on PrEP at community-level was needed and they provided several concrete recommendations on suitable methods.

Our data shows that PrEP is a largely unknown prevention tool, which remains ambivalent even when more information is provided. To understand this ambivalence, we need an intersectional understanding of how gender, migration background and sexual orientation influences acceptance [30]. Our findings highlight how community norms and values on sexuality and HIV acted as a barrier to PrEP uptake, framing PrEP use as a social practice in addition to being an individual choice [31]. Social norms on sexuality related mostly to faithfulness and monogamy, and sexuality and HIV were considered taboo. Hence PrEP could be framed as unnecessary and inducing promiscuity. Previous studies have shown that HIV stigma is indeed high among SSA diaspora communities in Belgium [32, 33]. However, our findings also highlight that cultural norms are not as deterministic as may be assumed, as shown by several comments about individual preferences and that once people are properly informed, acceptance will increase. This was also shown by a similar study in France [15]. Although the provision of information and PrEP promotion among sub-Saharan African diaspora can be considered a first step towards acceptance and potential uptake among this population, this alone is insufficient. This study has shown that the steps required to access PrEP were also perceived as a barrier. PrEP service delivery was deemed high threshold, which might withhold people from actually coming forward to PrEP, showing a need for simplified PrEP delivery. This was also shown by our previous studies [27]. Lowering the threshold for PrEP care, for instance by incorporating community-based organisations or family physicians, might be one way to do this [29]. However, broader structural issues will not be solved by this alone.

Secondly, the role of racism and discrimination has not been previously identified in relationship to PrEP. Our findings broadly confirm other studies’ results in this area, linking racism and discrimination to health outcomes, and conceptualizing racism as a social determinant of health [34–37]. Although participants did not relate this directly to PrEP acceptance, racism and discrimination may reduce people’s trust in healthcare services. Feeling targeted by HIV prevention yet being left out of PrEP promotion may seem contradictory, however it reiterates the same feeling of otherness because of an African heritage or being black in a predominantly white country. As one of our CRs commented during the data analysis, this may re-affirm the perception of not belonging to the mainstream ethnic groups. Trust and representation have historically not been built between public agencies, health care services and the SSA communities. The same may be true for research, which could partly explain the research gap on PrEP within these groups.

To effectively raise PrEP awareness and enabling this group that feels left behind to take informed decisions, we can build further on what has already been proven to be effective in HIV prevention or promoting of HIV testing. Using existing prevention structures, PrEP should be presented as a positive sexual health promotion tool [38], rather than only a risk reduction tool by community-based prevention projects that have already established trustful relationships with diaspora communities. Tailored awareness-raising campaigns should always address the specific knowledge gaps, but should be delivered within a comprehensive and holistic context of HIV de-stigmatization [32, 33]. Tailored approaches should be used for specific subgroups, such as LGBTQI+, in collaboration with existing organisations. Additionally, the use of opinion-leaders was found useful in general HIV prevention and could be useful in PrEP promotion as well [39–41]. Finally, to reach those SSA diaspora communities who are not reached by other efforts, interventions should involve community outreach workers to deliver information on sexual health promotion including PrEP [39–41]. Rigorous research in close collaboration with communities to evaluate such interventions is also needed.

### Limitations

Our study has several limitations. First, although we started from the assumption that community-led GDs would create a safer space for participants to express their opinions and concerns, implicit community norms may have influenced participant’s openness and freedom to voice their concerns and questions due to stigma or peer pressure. We also recruited participants on a voluntary basis, some already had existing relationships with community researchers, which may have made them more at ease to participate or may have impeded them in sharing private details. Additionally, next to having insiders of the communities (CRs), the presence of a researcher not member of the diaspora (EVL) might have affected participation during GDs. We countered this by clearly emphasising confidentiality, and taking a friendly, open and curious attitude during the GDs. However, having an outsider present could also have been perceived as an opportunity for the participants to voice their concerns considering ethnic targeting and discrimination, as participants have explicitly highlighted how experiences differ between black and white people. Additionally, since the socio-demographic questionnaire had open questions and was self-reported, some questions were left empty. We therefore miss some personal data on our participants.

## Conclusion

This study set out to better understand the community-level perceptions of PrEP among Sub-Saharan African diaspora in Belgium showing gaps in PrEP information and access. Our study highlights the urgent need for further accurate and tailored provision of PrEP information, thus enabling more people to make informed choices about HIV prevention methods and ultimately increasing their sexual agency, while also addressing health-care related and structural barriers to PrEP access and uptake, if we are to increase uptake among this population.

Statements and declarations.

## Data Availability

The data presented in this article are not readily publicly available because they contain information that could compromise the privacy of our research participants. A list of condensed meaning units or codes can be made available upon reasonable request to the corresponding author.

## Footnotes

1 The term “Sub-Saharan African migrant and diaspora communities in Belgium” has been chosen in consultation with our community researchers, as this aligns best with the self-definition of our study population. For readability, we have chosen to refer to this group as SSA diaspora for the remainder of this paper.
